# Hypozincemia Is Associated With Increased Tyrosine Levels, Low Handgrip Strength, Increased Incidence of HCC, and Liver Disease Progression—A Cross‐Sectional Study

**DOI:** 10.1002/jgh3.70124

**Published:** 2025-03-18

**Authors:** Tomoo Kobayashi, Jun Inoue, Yu Tanaka, Mitsuru Yamakawa, Makoto Kurihara, Tomoko Handa, Yutaka Kondo, Akihiro Saitou, Manabu Shiraki, Yasuhiro Kojima, Motoki Ohyauchi, Atsushi Masamune

**Affiliations:** ^1^ Department of Gastroenterology Tohoku Rosai Hospital Sendai Japan; ^2^ Division of Gastroenterology Tohoku University Graduate School of Medicine Sendai Japan; ^3^ Institute for Excellence in Higher Education Tohoku University Sendai Japan; ^4^ Medical Information Management Division Tohoku Rosai Hospital Sendai Japan

**Keywords:** chronic liver disease, handgrip strength, tyrosine, zinc

## Abstract

**Background:**

Serum zinc levels decrease in chronic liver disease (CLD), but their effects on liver reserve function, tyrosine, skeletal muscle mass, handgrip strength (HGS), and hepatocellular carcinoma (HCC) development remain poorly understood.

**Methods:**

A retrospective, cross‐sectional study was conducted on 516 CLD cases. Patients were divided into a low zinc group (< 80 μg/dL) and a high zinc group (≥ 80 μg/dL). Serum zinc levels were analyzed with liver reserve function (assessed by modified albumin‐bilirubin [mALBI] grade), tyrosine, branched‐chain amino acid/tyrosine ratio (BTR), and HCC development. In 180 cases, the relationship between serum zinc levels and skeletal muscle characteristics, including sarcopenia and HGS, was investigated.

**Results:**

Tyrosine levels increased significantly with mALBI grade progression. Patients in the low zinc group had higher tyrosine levels (76.9 vs. 67.2 μmol/L, *p* < 0.001), a greater proportion of high tyrosine levels (5.3% vs. 1.7%, *p* < 0.001), and more HCC cases (10.5% vs. 3.7%, *p* < 0.005). Zinc levels were lower with more severe CLD (81 μg/dL [mALBI grade 1] vs. 35.2 μg/dL [grade 3], *p* < 0.001). Tyrosine levels were higher in HCC patients than in non‐HCC patients (93.1 vs. 70.7 μmol/L, *p* < 0.001). Sarcopenia prevalence did not differ between groups (56.6% vs. 52.0%, *p* = 0.344), but low HGS was more frequent in low zinc patients (61.2% vs. 46.3%, *p* = 0.032). In a subset of patients with low zinc levels (*n* = 12), zinc supplementation reduced tyrosine levels after 3 months (86.3 vs. 73.3 μmol/L, *p* = 0.017).

**Conclusion:**

Hypozincemia is linked to elevated tyrosine levels, reduced HGS, increased HCC incidence, and CLD progression.

**Abbreviations**
AAA: aromatic amino acid
AIH: autoimmune hepatitis
ALBI: the albumin‐bilirubin
ALD: alcohol‐related liver disease
ALT: alanine aminotransferase
AST: aspartate aminotransferase
BCAA: branched‐chain amino acid
BTR: BCAA/tyrosine ratio
CT: computed tomography
HBV: hepatitis B virus
HCC: hepatocellular carcinoma
HCV: hepatitis C virus
MASLD: metabolic dysfunction‐associated steatotic liver disease
PBC: primary biliary cholangitis
PMI: psoas muscle index
SMM: skeletal muscle mass

## 1 Introduction

Zinc is a necessary cofactor for various enzymatic reactions [1]. Zinc homeostasis is primarily regulated in the liver, and chronic liver damage disrupts zinc homeostasis, resulting in zinc deficiency [2]. Hepatic inflammation with elevated levels of cytokines such as IL‐6 and TNFα caused by hepatitis C virus (HCV) has been reported to reduce zinc levels [3]. Serum zinc concentrations correlate with serum albumin levels [3, 4] and also correlate with the severity of symptoms, with levels below 50 μg/dL associated with more severe manifestations [1]. Zinc is also involved in ammonia metabolism and is associated with hepatic encephalopathy [1]. Zinc deficiency contributes to several other metabolic abnormalities in patients with chronic liver disease (CLD), including insulin resistance, fatty liver, and iron overload [2].

Regardless of the etiology, CLD, particularly liver cirrhosis, is associated with a deficiency of BCAAs and an increase in aromatic amino acids (AAAs) such as tyrosine [5, 6, 7]. This results in a decreased Fischer ratio, the ratio of BCAAs to AAAs [8]. The ratio of BCAAs to tyrosine (BTR) decreases as CLD progresses, making it a useful marker for evaluating amino acid imbalances [9]. In patients with cirrhosis, skeletal muscle mass decreases because ammonia is metabolized in the urea cycle using BCAAs in skeletal muscle. Ornithine transcarbamylase, an essential enzyme in the urea cycle, is a zinc‐dependent enzyme, suggesting a role for zinc in amino acid metabolism in muscles [5]. Serum zinc levels reflect protein synthesis capacity and are an independent predictor of reduced handgrip strength (HSS) and the development of sarcopenia [4]. AAAs increase due to portosystemic shunting and decreased liver reserve function [10]. Sarcopenia is a progressive loss of skeletal muscle volume associated with aging, leading to reduced muscle strength and poor prognosis in patients with liver cirrhosis [8]. Sarcopenia may result from decreased muscle protein synthesis, with deficiencies of essential amino acids, including BCAAs, contributing to these processes [8]. Zinc supplementation may reduce the consumption of BCAAs in muscle, allowing administered BCAAs to be used for albumin synthesis, which can lead to increased serum albumin levels and decreased ascites [11].

To date, cross‐sectional studies investigating the relationships among zinc, liver reserve, AAA, sarcopenia, and hepatocellular carcinoma (HCC) development are lacking. The aim of this study is to elucidate the impact of zinc on CLD by examining the relationship between zinc, modified albumin‐bilirubin grade (mALBI), and tyrosine. In addition, the effect of zinc on skeletal muscle mass (SMM) and HSS was analyzed in a subset of cases. The effect of zinc supplementation on amino acid imbalances was also evaluated in a small number of patients.

## 2 Methods

### 2.1 Study Subjects

This study was based on a retrospective, cross‐sectional analysis. Among 721 cases with liver disease who visited Tohoku Rosai Hospital and Tohoku University Hospital between January 1, 2022 and March 31, 2023, those without CLD or with insufficient data for evaluation were excluded. As a result, a total of 516 patients (240 males, 276 females) with CLD of various etiologies were included. CLD was defined based on findings such as blunted liver margins, changes in liver morphology, or coarse internal hepatic echoes observed on abdominal ultrasonography. Chronic hepatitis C was diagnosed by positivity for HCV RNA, and all cases with chronic hepatitis C in this study had undergone antiviral treatment resulting in HCV RNA negativity. These cases were included because liver fibrosis and hepatic reserve impairment may be present even after HCV RNA becomes undetectable. Chronic hepatitis B virus (HBV) infection was defined as being positive for HBs antigen for more than 6 months. Alcohol‐related liver disease (ALD) was defined as CLD resulting from the consumption of more than 60 g of alcohol per day. Non‐alcoholic steatohepatitis was diagnosed based on the basis of histologic findings [12] and was conveniently referred to as metabolic dysfunction‐associated steatotic liver disease (MASLD). Primary biliary cholangitis (PBC) and autoimmune hepatitis (AIH) were diagnosed clinically based on histologic diagnostic criteria from liver biopsy specimens [13, 14]. A total of 111 cases were diagnosed as liver cirrhosis based on abdominal ultrasonography, clinical examination, and liver histology (Table 1).

**TABLE 1:** Patient characteristics.

	HCV	HBV	ALD	MASLD	AIH	PBC	Others	Total
Number of patients (%)	181 (35.2%)	105 (20.3%)	58 (11.2%)	81 (15.7%)	49 (9.5%)	31 (6.0%)	11 (2.1%)	516 (100%)
Male/female	89/92	62/43	39/19	28/53	8/41	7/24	7/4	240/276
Age (years)	70.0 ± 11.0	61.9 ± 12.6	60.4 ± 11.9	61.3 ± 15.4	61.7 ± 14.0	64.1 ± 11.8	62.4 ± 13.9	64.7 ± 13.2
Skeletal muscle measurement (male/female)	48/49	10/2	19/7	13/24	1/2	1/2	1/1	93/87
Cases with low serum zinc levels (%)	89 (49.2%)	52 (49.5%)	41 (70.7%)	34 (42.0%)	20 (42.0%)	32 (64.5%)	7 (63.6%)	275 (53.3%)
Cases with cirrhosis (%)	21 (18.9%)	16 (14.4%)	31 (28.0%)	13 (11.7%)	11 (9.9%)	15 (13.5%)	4 (3.6%)	111 (100%)
Total bilirubin (mg/dL)	0.74 ± 0.71	0.82 ± 0.07	1.89 ± 2.69	0.85 ± 0.57	0.86 ± 0.71	0.85 ± 0.14	0.93 ± 0.71	1.16 ± 0.93
AST (IU/L)	26.0 ± 79.9	37.5 ± 303	66.9 ± 174.7	45.3 ± 34.7	49.1 ± 18.4	34.8 ± 0.71	37.3 ± 4.24	38.4 ± 44.0
ALT (IU/L)	23.0 ± 17.7	38.9 ± 455	55.5 ± 89.8	57.4 ± 142.1	53.5 ± 68.6	29.6 ± 11.3	33.2 ± 1.41	37.9 ± 52.2
Albumin (g/dL)	4.22 ± 0.78	4.22 ± 0.21	3.75 ± 0.99	4.22 ± 0.07	3.90 ± 0.92	3.96 ± 0.28	3.74 ± 0.50	4.11 ± 0.49
Platelet (×10^4^/μL)	18.8 ± 11.3	18.8 ± 2.05	16.8 ± 20.5	20.5 ± 12.1	21.8 ± 0.07	20.7 ± 5.16	18.4 ± 15.7	19.2 ± 6.87
Prothrombin (%)	97.4 ± 18.6	100.2 ± 24.7	81.5 ± 12.2	99.1 ± 25.7	100.1 ± 14.8	102.4 ± 3.11	87.9 ± 6.29	96.9 ± 20.7
Zinc (μg/dL)	78.6 ± 26.9	79.4 ± 4.24	65.0 ± 12.7	81.6 ± 2.83	73.4 ± 19.1	73.3 ± 2.83	72.4 ± 22.6	76.8 ± 14.5
AFP (ng/mL)	7.1 ± 107.6	17.4 ± 105.9	5.36 ± 0.71	5.18 ± 2.82	4.36 ± 6.08	5.01 ± 5.16	3.40 ± 0.07	8.23 ± 54.8
BCAA (μmol/L)	428.4 ± 118.7	427.3 ± 97.7	407.1 ± 274.4	466.6 ± 25.1	408.8 ± 129.8	378.9 ± 55.3	438.4 ± 64.7	426.1 ± 94.4
TYR (μmol/L)	68.9 ± 29.9	69.5 ± 2.26	86.5 ± 11.6	73.3 ± 0.07	77.57 ± 29.9	70.55 ± 14.9	70.2 ± 14.3	72.4 ± 23.7
BTR	6.41 ± 3.06	6.35 ± 0.83	5.32 ± 3.39	6.59 ± 0.23	5.61 ± 0.29	5.69 ± 0.93	7.04 ± 2.50	6.20 ± 1.65
ALBI	−2.87 ± 0.88	−2.85 ± 0.16	−2.32 ± 1.16	−2.84 ± 0.20	−2.58 ± 0.92	−3.21 ± 0.32	−2.45 ± 0.72	−2.81 ± 1.27

Abbreviations: AIH, autoimmune hepatitis; ALBI, the Albumin‐Bilirubin; ALD, alcohol‐related liver disease; ALT, alanine aminotransferase; AST, aspartate aminotransferase; BCAA, branched‐chain amino acid; BTR, BCAA/tyrosine ratio; HBV, hepatitis B virus; HCV, hepatitis C virus; MASLD, metabolic dysfunction associated steatotic liver disease; PBC, primary biliary cholangitis.

### 2.2 Blood Tests and, Imaging Studies

Biochemical blood tests performed on the participants included total bilirubin, aspartate aminotransferase (AST), alanine aminotransferase (ALT), albumin, serum zinc concentration before supplementation, alpha‐fetoprotein (AFP), platelet count, prothrombin time, BCAAs (valine, isoleucine, leucine), AAAs (tyrosine), and BTR. Tyrosine levels were measured by an enzymatic method, with a normal range defined as 51–98 μmol/L. Hypertyrosinemia was defined as a tyrosine level greater than 98 μmol/L. In this study, we defined a serum zinc concentration of less than 80 μg/dL as low serum zinc concentration, following the definition adopted by Murata et al. [4].

Liver reserve was assessed using the albumin‐bilirubin (ALBI) score and mALBI grade as defined below. ALBI score: (log10 bilirubin (μmol/L) × 0.66) + (albumin (g/L) × −0.085); and the ALBI grade was defined by the score as follows: ≤ −2.60 = Grade 1, > −2.60 to ≤ −1.39 = Grade 2, > −1.39 = Grade 3. ALBI grade 2 was further divided into two subgrades (2a and 2b) using a previously reported cutoff value (ALBI score −2.270), and these were designated as the mALBI grade [15, 16]. For the screening and evaluation of HCC, abdominal ultrasound, abdominal computed tomography (CT), and abdominal magnetic resonance imaging (MRI) were used. The presence or absence of HCC was defined as cases diagnosed with HCC based on abdominal imaging performed at the time of serum zinc testing, or cases with a history of HCC prior to the serum zinc testing.

### 2.3 Evaluation of Skeletal Muscle

The evaluation of HSS and SMM was performed in a total of 180 cases, including 93 males and 87 females. For these cases, muscle strength was assessed using HSS according to the sarcopenia criteria for liver diseases set by the Japan Society of Hepatology. The HSS cutoff values for diagnosing sarcopenia in liver disease are 28 kg for men and 18 kg for women. Similarly, the SMM was evaluated using the CT scan at the level of the third lumbar vertebra, calculating the psoas muscle area (cm^2^) per square meter of height squared (m^2^) (psoas muscle index, PMI) [17, 18]. The cutoff values for SMM reduction according to PMI are 6.36 cm^2^/m^2^ for men and 3.92 cm^2^/m^2^ for women.

### 2.4 Cases Administered With Zinc Acetate

Among the subjects of this study, 12 cases with low serum zinc levels (8 males and 4 females) were administered 50 mg/day of zinc acetate. The fluctuations in serum zinc and AAA tyrosine levels were examined before the administration and 3 months after the administration.

### 2.5 Ethical Review

This research was conducted in accordance with the Declaration of Helsinki. The research protocol was approved by the Ethical Committee of Tohoku Rosai Hospital (Tohoku 2023: 45). This is a retrospective observational study in which patient consent was obtained via an opt‐out method.

### 2.6 Statistical Analysis

The correlation between zinc levels and ALBI grade, tyrosine levels, and BTR was evaluated using Pearson's correlation. The relationship between ALBI grade and the proportion of low‐zinc cases by liver disease type was compared using the Kruskal–Wallis test. Tyrosine levels, the incidence of HCC, HSS, and SMM were compared between the normal zinc group and the hypozincemia group using Fisher's exact test. The relationship between tyrosine levels and HCC development was assessed using the Mann–Whitney *U* test. Changes in tyrosine levels and serum zinc concentrations before and after zinc acetate administration were evaluated using the paired *t*‐test. Predictors of HCC development were assessed with logistic regression analysis. All statistical analyses were performed using IBM SPSS Statistics 23 (IBM Japan Ltd., Tokyo, Japan).

## 3 Results

### 3.1 Relationship Between Chronic Liver Disease and Serum Zinc Levels

**FIGURE 1:**
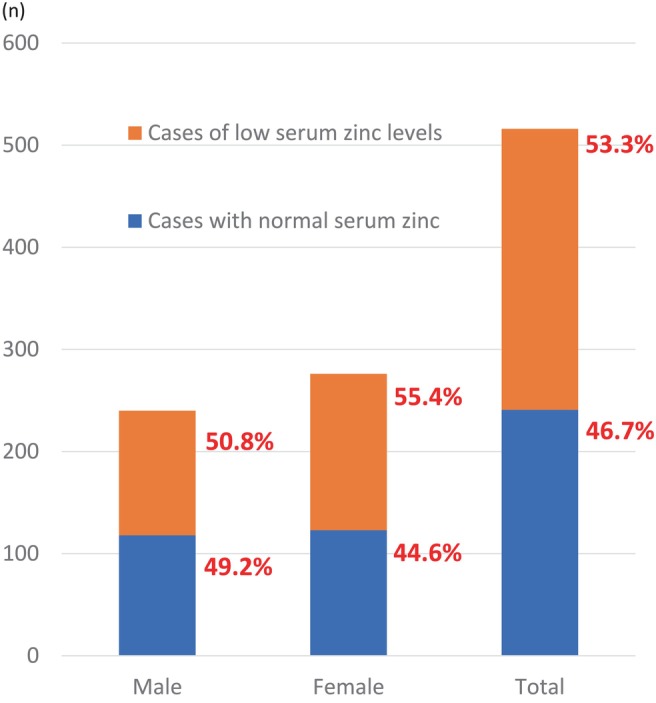
Percentages of patients with normal serum zinc levels and those with low levels in the subject cases.

**FIGURE 2:**
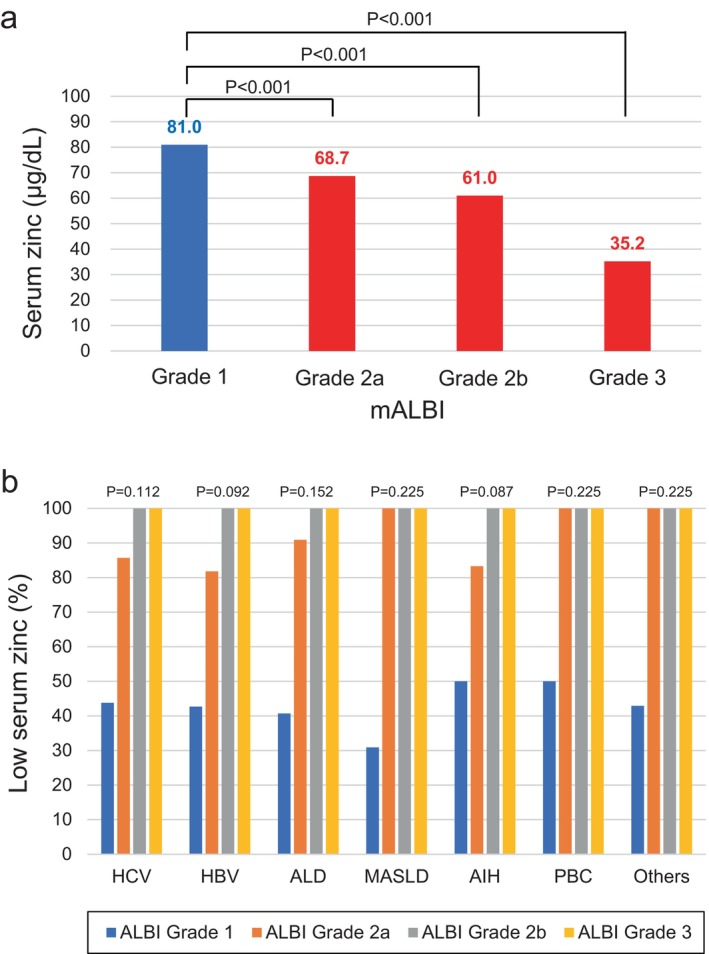
Analysis of the serum zinc levels in patients with chronic liver diseases according to the mALBI grade. (a) The mean values of serum zinc according to the mALBI grade in the overall cases. (b) The percentages of cases with low serum zinc levels (< 80 μg/dL) according to the underlying liver disease and the mALBI grade.

The characteristics of the study participants are shown in Table 1. Among them, 122 males (50.8%), 153 females (55.4%), and 275 patients overall (53.3%) had low serum zinc levels (Figure 1). Analysis of the relationship between ALBI grade and serum zinc levels showed a significant decrease in serum zinc levels as ALBI grade progressed (Figure 2a). Similarly, the proportion of low zinc cases increased with increasing ALBI grade in all types of CLD (Figure 2b).

### 3.2 Relationship Between Serum Zinc and Tyrosine Levels

**FIGURE 3:**
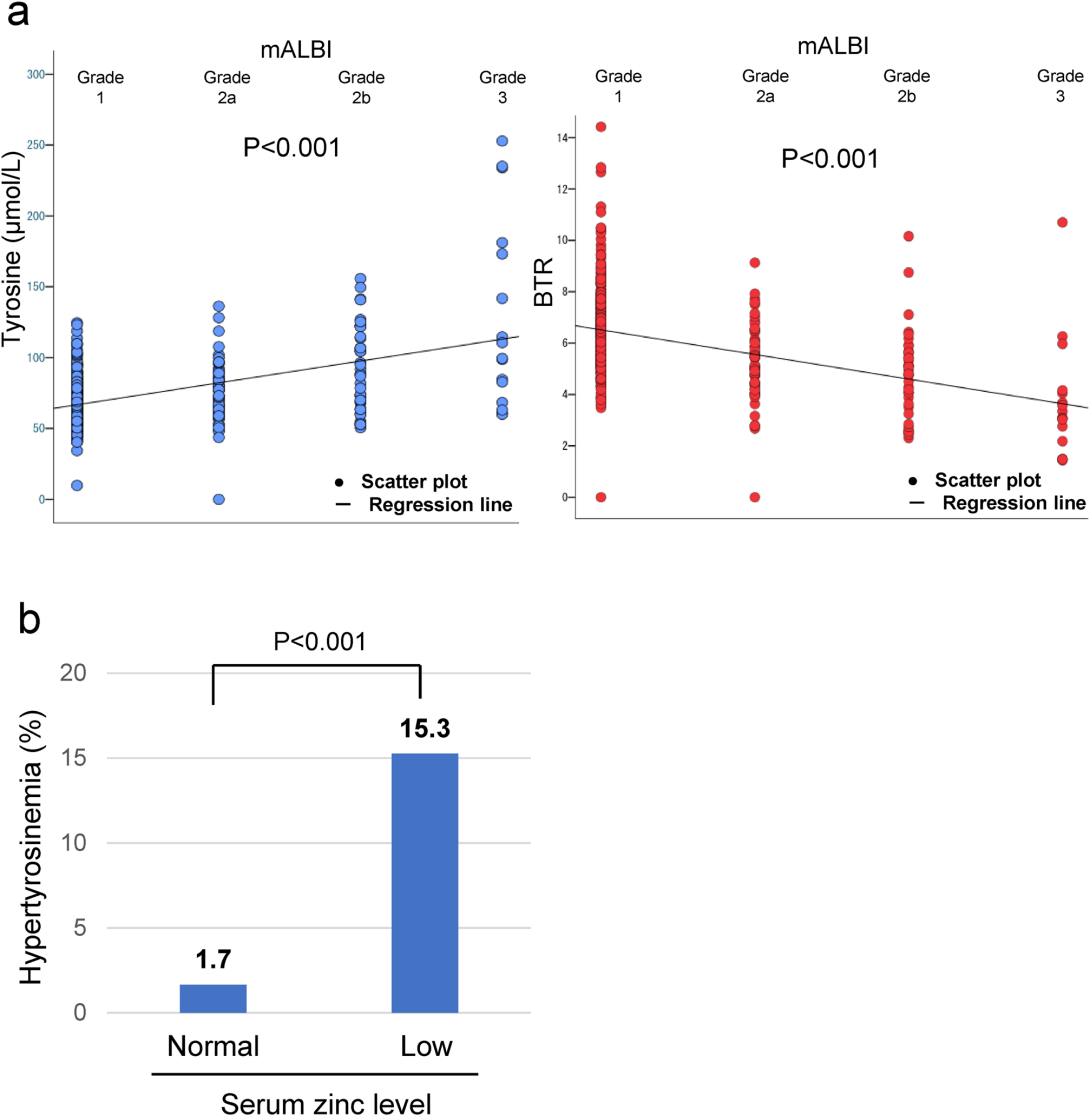
Analysis of serum tyrosine levels in patients with chronic liver diseases. (a) The relationship between the tyrosine levels and the mALBI grade, BTR and the mALBI grade. (b) Comparison of the percentage of cases with hypertyrosinemia between cases with normal zinc levels and those with low zinc levels.

Tyrosine levels increased significantly with increasing ALBI grade (*p* < 0.001) (Figure 3a). Conversely, BTR significantly decreased with increasing ALBI grade (*p* < 0.001) (Figure 3a). Hypertyrosinemia was observed in 4 out of 241 cases (1.7%) in the normal serum zinc group and in 42 out of 275 cases (15.3%) in the low serum zinc group. Low zinc group patients had high tyrosine levels (76.9 μmol/L vs. 67.2 μmol/L, *p* < 0.001). The proportion of hypertyrosinemia was significantly higher in patients with low serum zinc levels (*p* < 0.01) (Figure 3b).

### 3.3 Relationship Between Serum Zinc Levels and Handgrip Strength/Skeletal Muscle Mass

**FIGURE 4:**
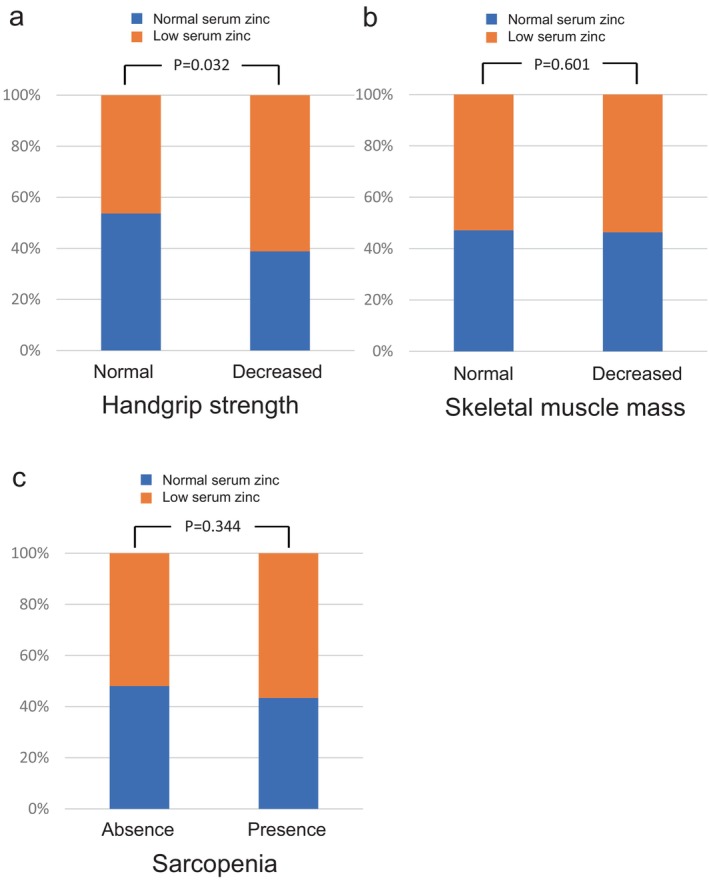
Comparison of the proportion of cases with normal and low serum zinc levels in patient groups separated according to muscle‐related parameters. (a–c) Comparison between cases with decreased handgrip strength and those without (a), comparison between those with decreased skeletal muscle mass and those without (b), and comparison between those with sarcopenia and those without (c) are shown.

Of the 180 cases in which SMM and HSS were measured, 85 patients had HSS below the reference values, of whom 52 (61.2%) had low serum zinc levels. The proportion of low serum zinc cases was significantly higher in the low HSS group (Figure 4a). A total of 110 patients had SMM below the reference values, including 59 cases (53.6%) with low serum zinc levels. However, there was no significant difference in the proportion of low serum zinc cases between the maintained and reduced SMM groups (Figure 4b). Fifty‐three patients met the criteria for sarcopenia as defined by the Japan Society of Hepatology, with 30 cases (56.6%) having low serum zinc levels. There was no significant difference in the proportion of low serum zinc cases between patients with and without sarcopenia (Figure 4c).

### 3.4 Relationship Between Serum Zinc Levels and HCC Development

**FIGURE 5:**
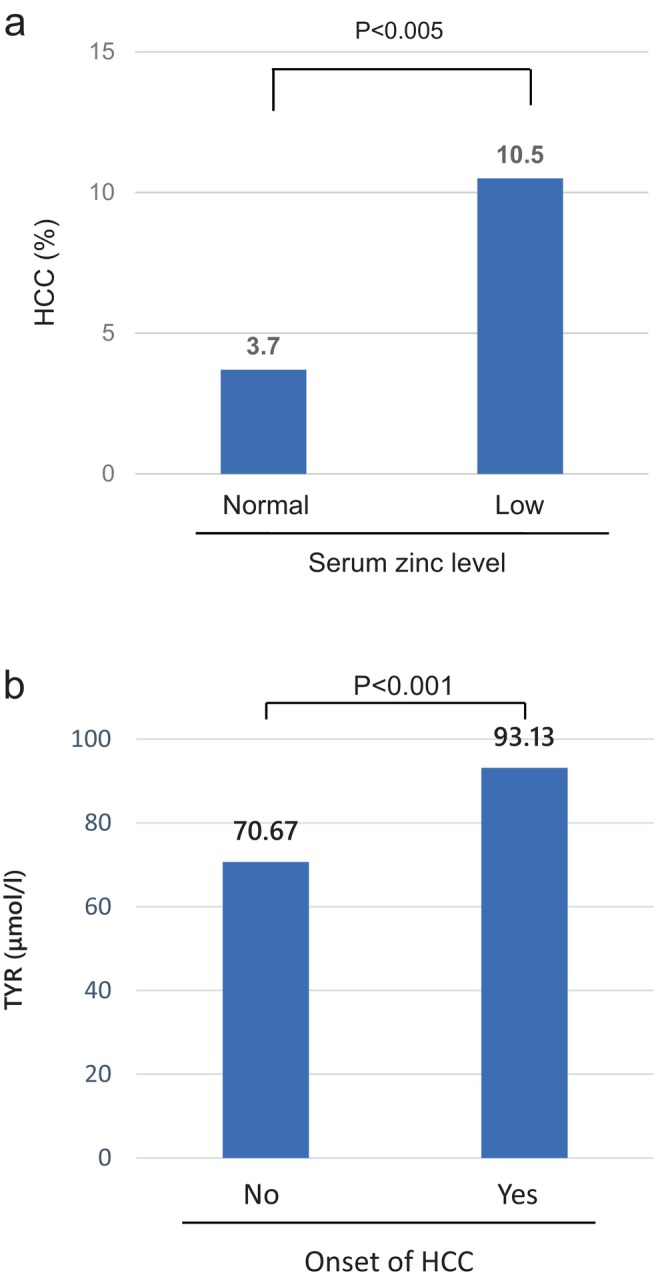
Serum zinc and tyrosine levels in cases of HCC onset. (a) Comparison of the incidence of HCC between cases with normal zinc levels and those with low zinc levels. (b) Comparison of tyrosine levels based on the presence or absence of HCC onset.

Among the 516 patients, HCC was identified in 38 cases (23 males and 15 females). Of these, 34 cases had a history of HCC at the time of zinc data collection, and 4 cases developed HCC during a mean follow‐up period of 15 months after zinc data collection. Of the 38 HCC cases, 29 (76.3%) had low serum zinc levels, including 18 males and 11 females. All four newly diagnosed HCC cases had low serum zinc levels at baseline. In the normal serum zinc group, 9 out of 241 cases (3.7%) had HCC, whereas in the low serum zinc group, 29 out of 275 cases (10.5%) had HCC, showing that the incidence of HCC was significantly higher in the low serum zinc group (*p* < 0.01) (Figure 5a). During the observation period, 12 patients died; 10 deaths were due to HCC, and 2 deaths were due to liver failure. All patients who died had low serum zinc levels. The tyrosine concentration in the HCC group was significantly higher at 93.13 μmol/L compared with 79.56 μmol/L in the non‐HCC group (Figure 5b). Univariate logistic regression analysis identified mALBI, low serum zinc levels, and high tyrosine levels as factors associated with HCC development. However, in the multivariable logistic regression analysis, only mALBI was significant (*p* < 0.001). Each one‐grade increase in mALBI was associated with a 2.302‐fold increased risk of HCC development (Table 2).

**TABLE 2:** Factors involved in the development of hepatocellular carcinoma.

Factors	Significant probability (univariate)	Significant probability (multivariate)	B	Exp(B)
mALBI	0.0000	0.0000	0.834	2.302
Zinc	0.0000	—	—	—
TYR	0.0000	—	—	—
Etiology of liver disease	0.3118	—	—	—
PMI	0.3285	—	—	—
Grip strength	0.7824	—	—	—
Sarcopenia	0.7125	—	—	—
Gender	0.3918	—	—	—

Abbreviations: B, partial regression coefficient; Exp (B), odds ratio.

### 3.5 Evaluation of 12 Cases Treated With Zinc Acetate

**FIGURE 6:**
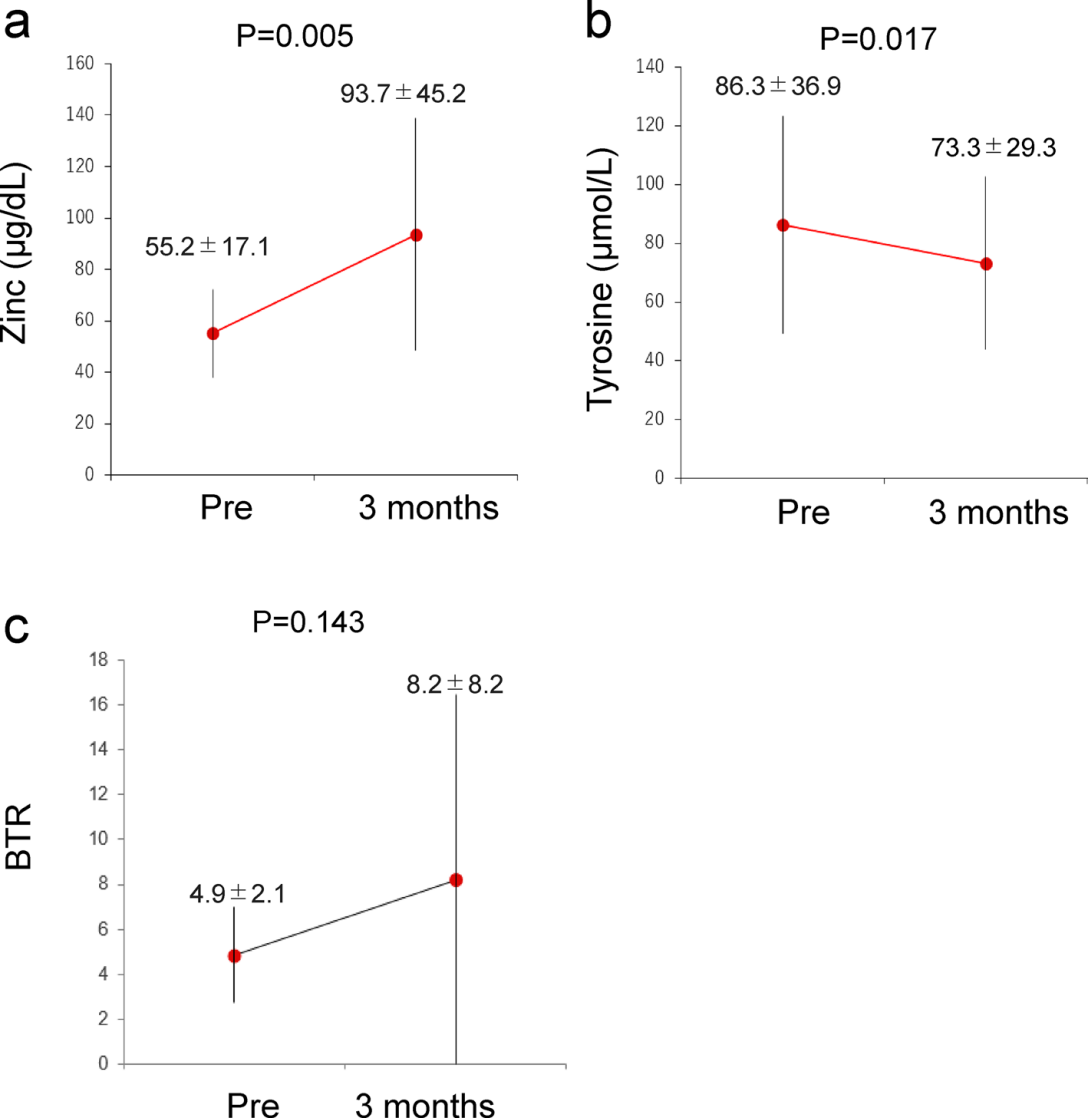
Effects of zinc acetate administration (*n* = 12). (a–c) Changes in serum zinc (a), tyrosine (b), and BTR (c) before and 3 months after zinc acetate administration are shown. Dots and bars indicate means and standard deviations.

Twelve cases with serum zinc levels below 80 μg/dL were treated with oral zinc acetate (50 mg/day). After 3 months, serum zinc levels increased significantly (Figure 6a) and tyrosine concentrations decreased significantly (*p* < 0.05) (Figure 6b). Although the sample size was small, the results suggest that zinc acetate treatment may improve amino acid imbalances.

## 4 Discussion

This study focused on serum zinc levels and tyrosine concentrations associated with disease progression in CLD. The main results of this study are as follows: (1) Tyrosine levels increased significantly with the progression of ALBI grade, and the number of cases with low serum zinc levels also increased significantly. High tyrosine levels were significantly more common in the low serum zinc group. (2) In the group with HCC, there were significantly more cases with low serum zinc levels and higher tyrosine levels than in the group without HCC. (3) The group with reduced HSS had a significantly higher proportion of cases with low serum zinc levels. (4) In a small number of cases with low serum zinc levels, a 3‐month course of zinc acetate resulted in a significant reduction in tyrosine levels.

Zinc deficiency in cirrhosis is thought to be caused by decreased zinc absorption in the gastrointestinal tract, increased urinary zinc excretion due to diuretic use, nutritional deficiencies, hypoalbuminemia, and portal‐systemic shunting [5]. Zinc absorbed in the intestines travels in the bloodstream bound to albumin [3]. The decrease in zinc levels associated with the progression of ALBI grade is thought to be due to these underlying conditions. The mechanism behind the increase in tyrosine levels with the progression of ALBI grade remains unclear.

In cirrhosis caused by HCV, risk factors for the development of HCC have been reported to include zinc deficiency, high AFP levels, low BTR, and male gender [19]. In that study, zinc deficiency was identified as the only significant predictor of HCC development in multivariate Cox regression analysis, with a hazard ratio of 1.61 [19]. In this study, the HCC group had a higher proportion of cases with low serum zinc levels and exhibited hypertyrosinemia, but the only significant factor identified in the multiple logistic regression analysis was mALBI, indicating that the impact of liver function decline was greater. However, because zinc levels were strongly associated with mALBI, it was considered unlikely that the zinc levels would remain a significant factor after multivariate analysis.

Zinc deficiency has been reported to be an independent predictor of sarcopenia, which was defined as reduced HSS and reduced SMM [20]. In this study, the average mALBI score of the subjects was −2.81, which corresponds to ALBI grade 1, indicating that liver function was relatively well preserved (Table 1). For this reason, the significant difference in low zinc frequency could only be seen in reduced HSS (Figure 4). If more patients with cirrhosis were included, significant differences might be seen in other parameters.

Administration of zinc acetate to cases with low serum zinc levels may improve the plasma amino acid imbalance by reducing tyrosine levels, resulting in an increase in the BTR. It has been reported that, in the treatment of nitrogen metabolism abnormalities in liver cirrhosis, the combination of BCAAs and zinc significantly improves the rate of change of the Fischer ratio over a treatment period of 5–6 months compared to BCAAs alone (1.22 vs. 1.08, *p* = 0.0165) [21]. The addition of zinc acetate to BCAAs may help to further improve amino acid composition by increasing the BTR.

The strength of this study lies in the first demonstration that decreased serum zinc levels were associated with mALBI, high tyrosine levels, decreased HSS, and the development of HCC. Also, this study suggests that zinc supplementation may contribute to the improvement of amino acid imbalance first. In addition, this study has several limitations. First, this is a retrospective observational study in only two institutions. Second, the number of cases receiving zinc acetate was very small, suggesting that further prospective studies with a larger number of cases are needed.

In conclusion, this study demonstrated the association between the serum zinc levels and disease progression in several CLDs and suggested the involvement of amino acid imbalances, including tyrosine. Larger studies are needed to confirm the importance of zinc and the usefulness of its supplementation in CLDs.

## Conflicts of Interest

The authors declare no conflicts of interest.
